# Non-coding RNAs in osteoarthritis and osteoporosis-related hip fractures: molecular biomarkers for precision diagnosis and prognosis

**DOI:** 10.3389/fendo.2025.1653831

**Published:** 2025-10-13

**Authors:** Chengxuan Zhang, Yu Chen, Jian Zhang

**Affiliations:** ^1^ The First Clinical College of Chongqing Medical University, Chongqing, China; ^2^ Orthopedics Department, The First Affiliated Hospital of Chongqing Medical University, Chongqing, China

**Keywords:** hip fracture, osteoporosis, osteoarthritis, non-coding RNAs, biomarkers, diagnostics

## Abstract

As individuals age, bone density declines and the likelihood of fractures increases, particularly in women experiencing menopause. Hip fractures, the most severe consequence of osteoporosis, are becoming more common due to the aging global population and a 1-3% annual increase in hip fractures in most regions. The specific epigenetic mechanisms underlying the onset of osteoporosis and its related fractures remain predominantly unexamined. Research indicates that epigenetic modifications can elevate the risk of osteoarthritis, osteoporosis and bone fractures, especially hip fractures, by linking genetic predispositions with environmental factors. Essential regulatory components in these factors encompass microRNAs, lncRNAs, and circular RNAs. This review examines the function of miRNAs, lncRNAs, and circRNAs in the advancement of osteoporosis, with an emphasis on osteoblasts and osteoclasts. The objective is to enhance comprehension of RNA classes in osteoporotic hip fractures, which may facilitate early detection and prognosis, as well as clarify cellular interactions, potentially resulting in innovative diagnostic techniques and targeted therapies.

## 1 Introduction

Human bone metabolism encompasses resorption and formation, regulated by osteoclasts and osteoblasts. Osteoporosis, a metabolic disorder, is characterized by diminished bone density and increased resorption, thereby elevating the risk of fractures (1). Osteoporotic fractures predominantly occur in specific anatomical sites such as the hip, pelvis, spine, wrist, and forearm, with hip fractures being especially severe and debilitating. Hip fractures are a common source of disability, with annual mortality rates reaching 30%. Immutable risk factors encompass lower socioeconomic status, advanced age, female gender, previous fractures, metabolic bone disorders, and osseous malignancies. Alterable risk factors encompass low body mass index, osteoporosis, heightened fall risk, medications influencing fall risk or bone mineral density, and substance abuse (2–4). Postmenopausal women are predominantly susceptible to osteoporotic hip fractures, and existing diagnostic tools exhibit limitations, underscoring the necessity for novel early diagnostic methods and treatment protocols for osteoporosis (5, 6).

One common joint degenerative disease that affects adults and causes pain and disability is osteoarthritis (OA). Age, obesity, joint damage, and heredity are some of the factors that lead to its development. OA results in abnormalities of the meniscus, synovial tissue, subchondral bone, and articular cartilage. Degradation of cartilage, development of osteophytes, subchondral sclerosis, and hyperplasia of synovial tissue are important characteristics. Numerous molecules and pathways play a role in the pathophysiology of OA, with the NF-κB pathway being activated by systemic inflammation and secreted cytokines such as TNF-α and IL-1β. Current research emphasizes how non-coding RNAs (ncRNAs), such as circular RNAs, microRNAs, and long ncRNAs, regulate the development of OA (7). Furthermore, studies indicate that the advancement of osteoporosis is affected by epigenetic elements, exhibiting variations in miRNA, lncRNA, and circRNA expression in postmenopausal individuals relative to healthy controls (8, 9). This has resulted in heightened attention on miRNAs, lncRNAs, and circRNAs as prospective therapeutic targets or biomarkers. They can encode functional peptides that influence the activities of osteoblasts and osteoclasts by modulating cellular signaling, regulating gene expression, and participating in various biological processes via their small open reading frames (10–12). This review examines the functions of miRNAs, lncRNAs, and circRNAs in the progression of osteoarthritis and osteoporosis, their impacts on osteoblasts and osteoclasts, and their prospective uses as biomarkers and therapeutic targets.

## 2 The association between noncoding RNAs and osteoporosis

### 2.1 Noncoding RNAs

**Figure 1:**
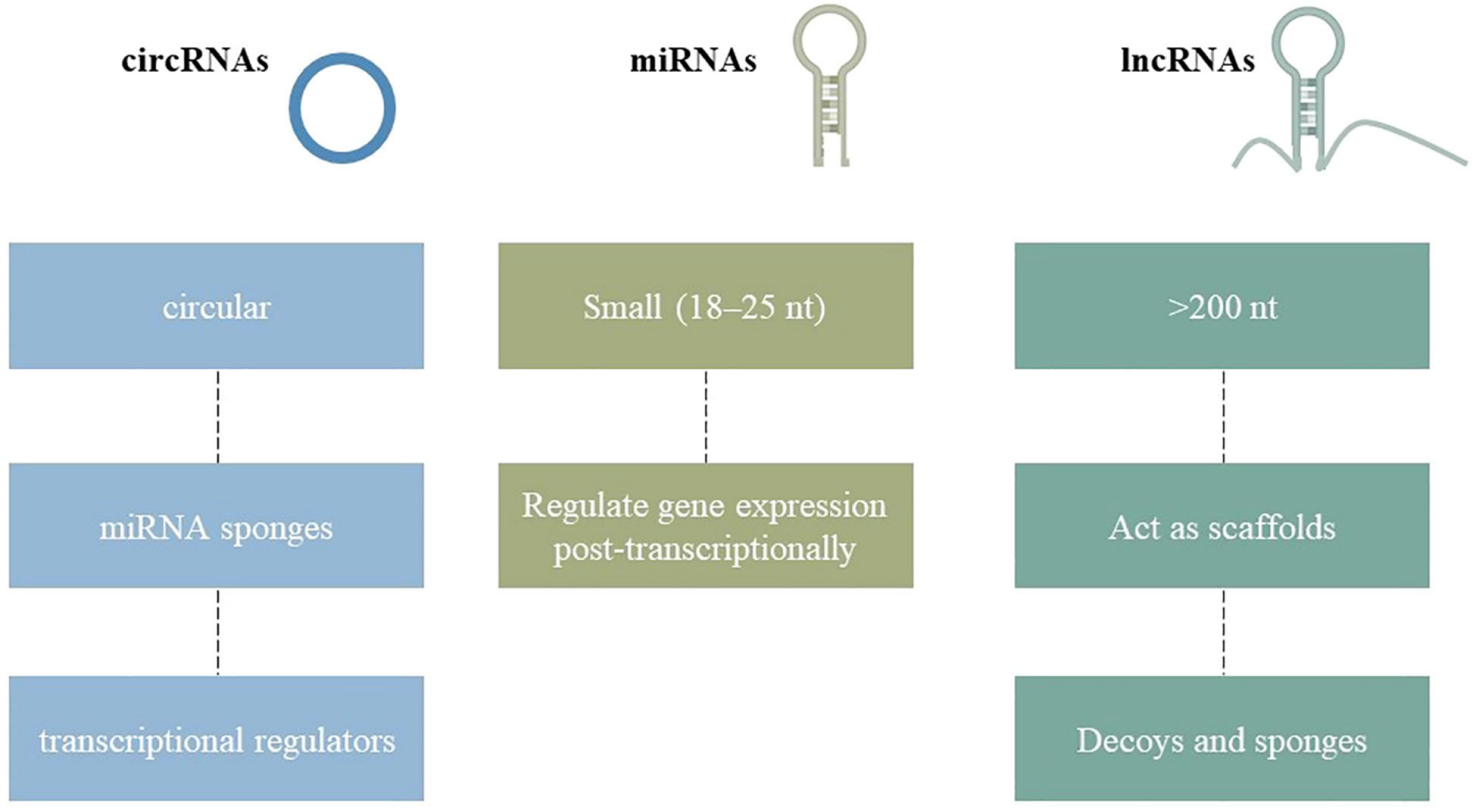
Structural diversity of non-coding RNAs (ncRNAs) relevant to osteoporosis. MicroRNAs are short linear sequences, long non-coding RNAs are extended transcripts with diverse regulatory functions, and circular RNAs form closed loops that often act as molecular sponges. These RNA classes collectively influence osteoblast and osteoclast activity, shaping bone health.

Non-coding RNAs (ncRNAs) are RNA molecules generated during genome transcription, encompassing miRNA, siRNA, circRNA, sncRNA, and lncRNA, which do not encode proteins. Long non-coding RNAs (lncRNAs), exceeding 200 nucleotides in length, modulate cellular processes such as proliferation, differentiation, and apoptosis, influencing gene expression, protein translation, and stability, while serving as decoys, scaffolds, or competing endogenous RNAs, which may contribute to disease pathogenesis (13). MicroRNAs, measuring 18–25 nucleotides in length, modulate post-transcriptional gene expression. Primary miRNAs (pri-miRNAs) represent the most rudimentary form, which is processed by RNA endonuclease III (Drosha) and DGCR8/Pasha to yield pre-miRNAs, a precursor of microRNAs, contributing to disease. MicroRNAs, which regulate cell proliferation, differentiation, morphogenesis, and apoptosis, are essential for sustaining or determining cell survival following cleavage by the enzyme Dicer (14). CircRNAs are non-coding RNAs characterized by a closed-loop structure, varying in length from 100 to several thousand nucleotides, generated via reverse splicing. They function as miRNA sponges or competing endogenous RNAs (ceRNAs), vying for miRNA binding and affecting transcription within the nucleus. CircRNAs are additionally associated with protein factors. Small non-coding RNAs, such as tRNA-derived small RNAs, snoRNAs, snRNAs, and PIWI-interacting RNAs, are predominantly examined in tumors, presenting a novel research opportunity concerning osteoporosis (**Figure 1**) (15).

### 2.2 The regulatory function of ncRNAs in osteoporosis

**Figure 2:**
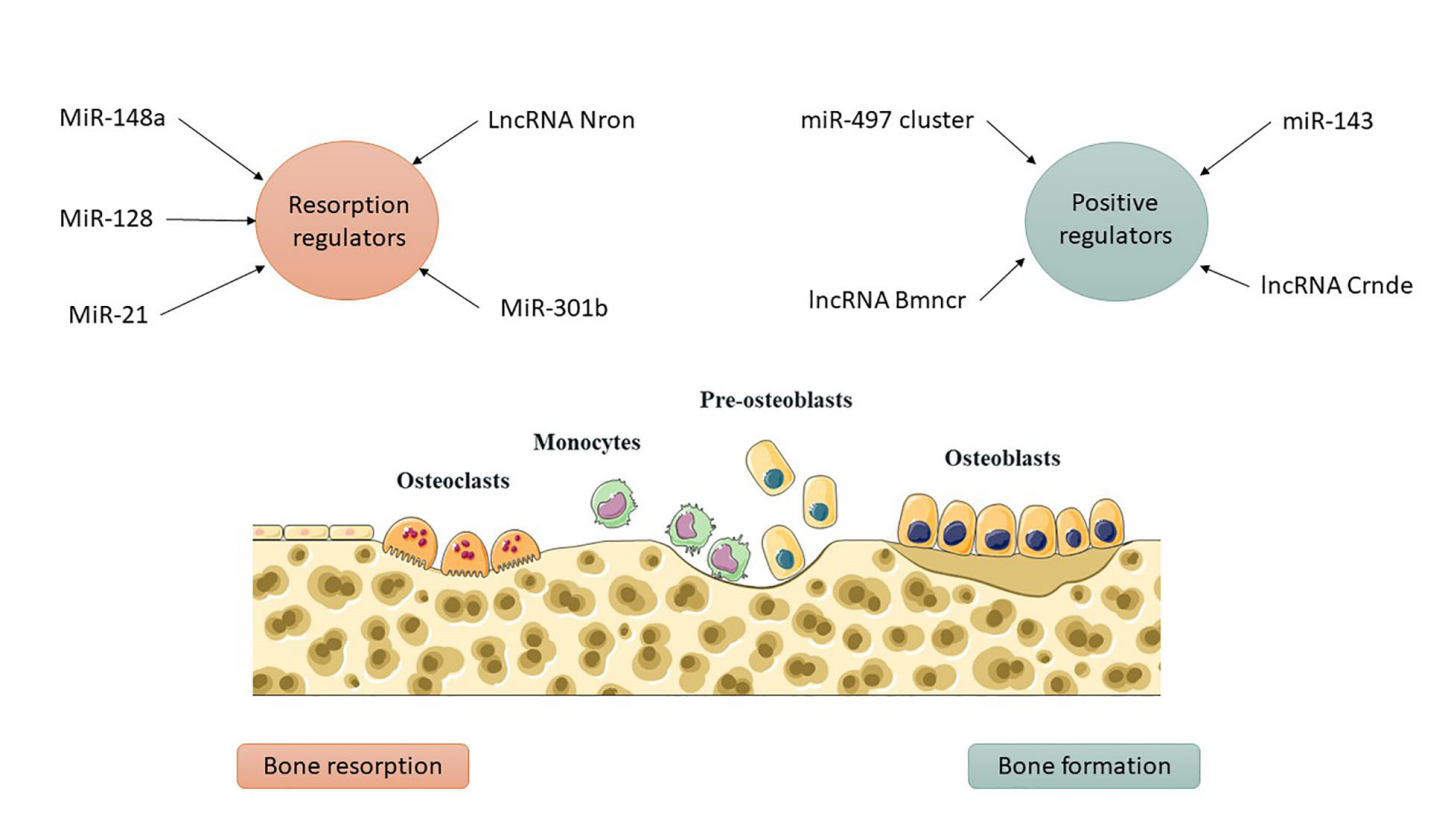
ncRNAs as regulators of bone remodeling. Osteoblasts and osteoclasts normally maintain a balance between bone formation and resorption. Dysregulated ncRNAs disrupt this balance, tipping it toward bone loss and increasing the risk of osteoporosis and fractures.

Bone metabolism encompasses both anabolism and catabolism, with osteoblasts and osteoclasts being essential for bone formation and resorption. Osteoporosis, defined by diminished formation and heightened absorption, elevates the risk of fractures (16). Recent studies have shown that ncRNAs play a pivotal role in osteoporosis, with over 300 dysregulated ncRNAs identified in postmenopausal individuals. Modulating these associated ncRNAs may be advantageous in the treatment of osteoporosis, with critical functions confirmed via genetic animal models (**Figure 2**) (17, 18). The research identifies functional bone ncRNAs associated with fractures and bone mineral density in postmenopausal women, delineating specific subsets in weight-bearing and non-weight-bearing skeletal locations. Seventy-five iliac bone ncRNAs and ninety-four femoral bone ncRNAs were identified as being associated with total hip bone mineral density (BMD), with five ncRNAs exhibiting a positive correlation with BMD in femoral bone (19).

A novel molecular mechanism implicated in the pathophysiology of osteoporosis has been elucidated by the identification of the LINC00339-PARP1-CDC42 regulatory axis. This finding raises the possibility that the LINC00339-PARP1 interaction could be a promising therapeutic target for altering osteoblast dysfunction, as well as expanding our understanding of how epigenetic regulation impacts bone remodeling. Importantly, these findings offer a conceptual framework for lncRNA-driven therapies in skeletal disorders and demonstrate the translational potential of RNA-protein complex targeting to restore bone homeostasis. A study employing high-throughput transcriptome sequencing and bioinformatics identified differentially expressed mRNAs and ncRNAs in bone mesenchymal stem cells from individuals with senile osteoporosis. The research identified substantial changes in 415 mRNAs, 30 lncRNAs, 6 circRNAs, and 27 miRNAs, with the proposed network possibly functioning as a therapeutic target (20).

Postmenopausal women frequently encounter osteoporosis as a result of estrogen deficiency, calcium depletion, and the aging process. A study identified a circRNA-associated ceRNA network in the bone marrow stem cells of ovariectomized mice, demonstrating diminished bone mass and heightened osteogenesis in OVX-induced osteoporotic mice. The research examined mRNAs within the circRNA-associated ceRNA network, possibly associated with osteoporosis pathology. Variably expressed mRNAs were identified as participating in extracellular matrix-receptor interactions, fatty acid metabolism, and PPAR signaling (21). The study identifies PPARγ, PI3K-Akt, and MiR-216a as modulators of osteoblast proliferation and differentiation (22). It also identifies potential ceRNAs implicated in osteoporosis, concentrating on circRNA-associated networks that regulate OP-related genes and pathogenesis. The data improves comprehension of circRNA-associated ceRNA networks in osteoporotic conditions (21).

Innovative circRNA networks, such as circRNA 0020 and circRNA 3832, represent prospective biomarkers for osteoporosis research. Non-coding RNA and Wnt signaling pathways are essential for the pathogenesis of osteoporosis, with miR-23b-3p suppressing conditions in murine models and miR-146a promoting differentiation in human mesenchymal stem cells (23). Comprehending these pathways will guide precise molecular therapy. Investigations into the CircRNA/Wnt axis are concentrating on its involvement in osteoporosis, facilitating osteogenic differentiation via the Wnt/β-catenin pathway, and augmenting osteogenesis through CircStag1. lncRNAs are pivotal in epigenetic modifications during mesenchymal stem cell (MSC) differentiation and degenerative bone diseases, modulating gene expression and possibly serving as biomarkers for diagnosis and prognosis (24). Nonetheless, obstacles such as off-target effects and intricate secondary structures necessitate advanced bioinformatic tools for precise interaction prediction and sequencing technologies.

## 3 Bone formation

### 3.1 Positive regulators

Osteoblasts, derived from mesenchymal stem/stromal cells, regulate bone homeostasis by facilitating bone formation. Research has demonstrated that MiR-143, a gene predominantly expressed in osteoblasts, is crucial for promoting osteoblast differentiation in osteoporosis (25). The overexpression of this factor was observed to impede osteoblastic differentiation in miR-143-deficient mice. The research employed mRNA sequencing, target prediction, and luciferase reporter assays. Injection of HDAC7-siRNA into miR-143 knockout mice resulted in substantial alleviation of symptoms. Administration of AgammiR-143 expedited osteogenesis and mitigated bone loss in a mouse model of aging-induced osteoporosis (26). The miR-497 cluster, which targets F-box and WD-40 domain proteins, sustains Notch activity and HIF-1α stability in endothelial cells (27). LncRNA Bmncr affects the fate of aging bone marrow stem cells, resulting in osteopenia in mice. Overexpression enhances bone density and stimulates bone formation via TAZ-ABL interaction (28). Knockout mice exhibit diminished osteoblast proliferation and bone mass attributed to decreased expression of Alp, Runx2, and Osx, highlighting the significance of Crnde in osteoblast proliferation and differentiation (29) (**Table 1**).

**Table 1:** The function of certain ncRNAs in osteoporosis and their mechanisms in bone formation.

NcRNAs	Targeting pathways	Molecular function	References
MiR-338	Runx2/Sox4/miR-338 signaling	The removal of the miR-338 cluster or the administration of a miR-338 cluster inhibitor inhibited osteoporosis following ovariectomy.	(30)
Neat1	Smurf1/Runx2	Neat1 deficiency in osteoblasts diminished their response to mechanical stimulation.	(31)
MiR-451a	Bmp6/SMAD1/5/8	Osteoblasts and mesenchymal stem cells from miR-451a knockout mice were observed to enhance osteogenesis.	(32)
MiR-146a-5p	Sirt1	MiR-146a-5p was identified as an inhibitor of osteoblast differentiation in BMSCs, and its absence safeguarded female mice from age-related bone loss.	(33)
MiR-143	HDAC7	Facilitating angiogenesis in conjunction with osteoblast differentiation	(26)
MiR-188	HDAC9 and RICTOR	Inhibition of miR-188 in aged mice resulted in enhanced bone formation and reduced accumulation of bone marrow adipose tissue.	(34)
MiR-146a	Not determined	It inhibited the proliferation and differentiation of osteoblasts while simultaneously promoting the apoptosis of mesenchymal stem cells (MSCs).	(35)
MiR-497 ~ 195 cluster	Notch and HIF-1a	Facilitating angiogenesis and osteogenesis is a goal for age-related osteoporosis.	(27)
Crnde	Wnt/β-catenin signaling	The knockout of Crnde markedly hindered osteoblast proliferation and differentiation.	(29)
Bmncr	Interaction between FMOD and TAZ RUNX2/PPARG	Restoring BMNCR levels in human BMSCs reversed the age-associated transition between osteoblast and adipocyte differentiation.	(20)
MiR-143/145	Sox2	Overexpression of miR-143/145 compromised the self-renewal and osteoblastic differentiation capabilities of BMSCs.	(36)
MiR-185	Bgn/BMP/Smad signaling	Excessive bone formation following the depletion of miR-185	(37)

### 3.2 Negative regulators

Certain non-coding RNAs have been identified as negative regulators of osteogenesis. Li and colleagues performed a miRNA microarray analysis to compare bone marrow stromal cells (BMSCs) in young and aged mice, discovering that miR-188 expression was markedly elevated in aged mice. The global knockout of miR-188 or the application of aptamer-antagomiR-188 in bone marrow diminished age-related bone loss and enhanced adipogenesis, whereas its overexpression in osterix+ osteoprogenitors or bone marrow-derived stem cells exacerbated bone loss and adipogenesis (34). MiR-188 targets HDAC9 and RICTOR, thereby augmenting osteoblastic activity and bone formation in murine models (34). Expression of MiR-146a-5p is elevated in the bone tissue of aging female mice and patients with postmenopausal osteoporosis (PMOP). MiR-146a−/− mice exhibited mitigated bone loss, expedited MSC proliferation, and diminished apoptosis, as indicated by elevated osteoblastic markers (38). Zheng et al. discovered that Sirt1 can interact with miR-146a-5p in its 3'-UTR, indicating its potential role in enhancing bone health. The protein expression of Sirt1 was markedly increased in miR-146a global knockout mice, indicating a correlation with aging and a possible role in osteoporosis prevention (33). LncRNA Neat1 was essential for osteoblastic differentiation, and its depletion compromised bone structure and resulted in loss in mice exposed to mechanical loading. Depletion of Neat1 impeded bone formation and mitigated unloading-induced bone loss. Paraspeckles enhance the nuclear retention of Smurf1 and inhibit translation, resulting in the degradation of Runx2 (39). PMOP is a prevalent bone disorder, with miR-143 and miR-145 identified as potential therapeutic targets. The overexpression of miR-143/145 impedes osteoblastic differentiation in bone marrow stem cells, whereas the knockout or use of antagomiR-143/145 enhances estrogen-deficient osteoporosis in female mice (40). Cytoplasmic miR-143/145 and lncRNA MIR143HG, regulated by ERβ, modulate the translation of pluripotency genes through the canonical ceRNA pathway. MIR143HG collaborates with miR-143 to jointly activate SOX2 transcription (40). Knockout of miR-185 diminishes bone loss in estrogen-deficient osteoporosis models, whereas miR-185 agomir reinstates bone formation. The enrichment of the MiR-338 cluster in PMOP patients and OVX-induced mice indicates that MiR-338-3p or miR-3065-5p may alleviate osteoporotic symptoms (37).

## 4 Bone resorption

**Figure 3:**
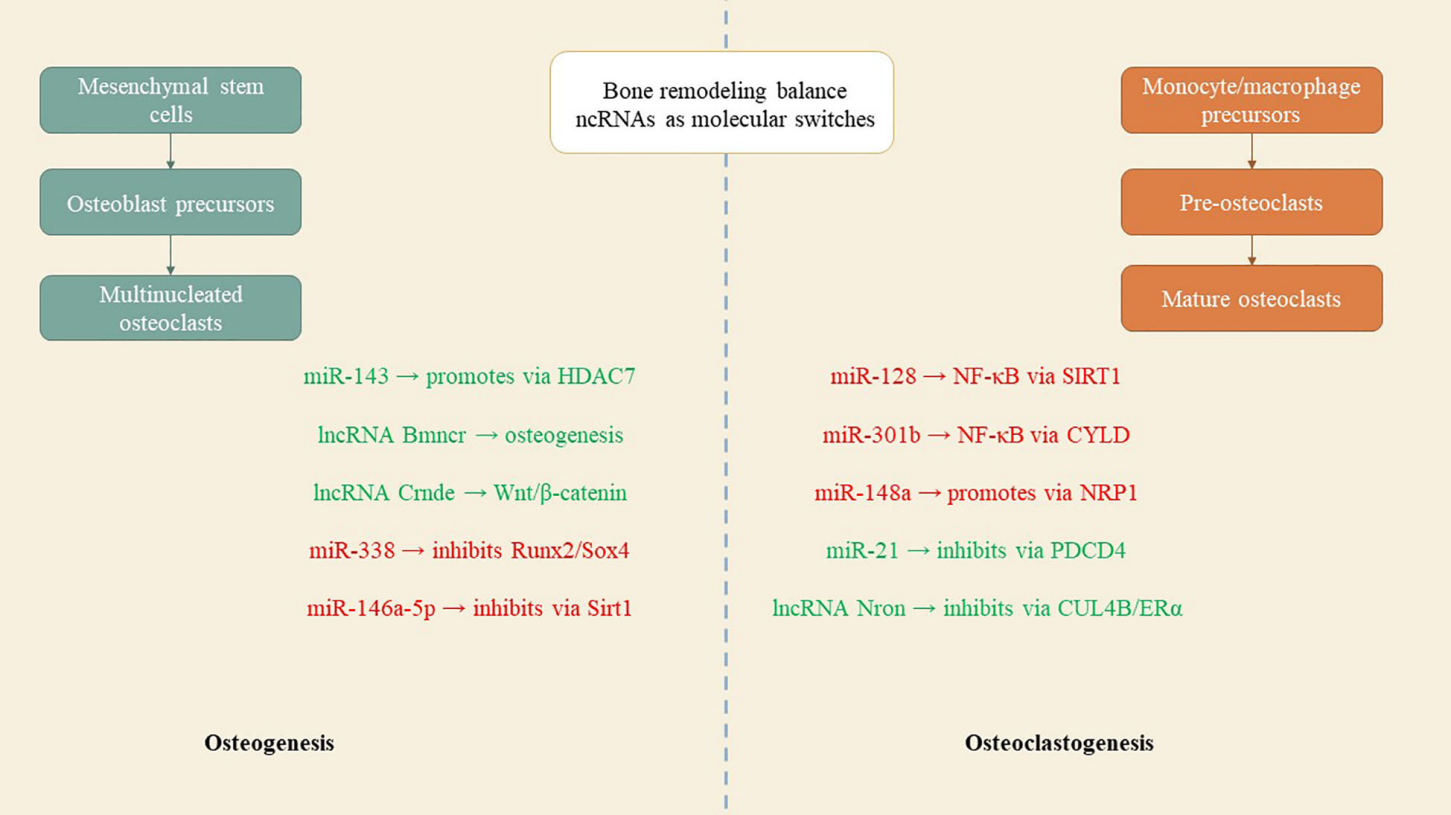
ncRNAs as molecular switches in bone metabolism. Positive regulators such as miR-143, lncRNA Bmncr, and lncRNA Crnde promote osteoblast differentiation, while inhibitory ncRNAs like miR-338 and miR-146a-5p suppress bone formation. On the resorption side, ncRNAs such as miR-128, miR-148a, and miR-301b enhance osteoclast activity, whereas miR-21 and lncRNA Nron counteract bone loss. Their interplay determines whether bone is preserved or degraded.

Comprehending the aberrant bone resorption by osteoclasts is essential for the diagnosis, treatment, and prevention of osteoporosis, given their distinction from macrophage/monocyte precursor cells affected by RANKL and other osteoclastogenic factors (37). Mir-21, an RNA molecule, modulates bone resorption in mice by inhibiting osteoclast formation, preserving skeletal structure, and alleviating osteoporosis, with its target PDCD4 influencing osteoclast differentiation (41). Mir-128, a crucial RNA in aging and inflammatory disorders such as osteoporosis, facilitates osteoclastogenesis in postmenopausal individuals and ovariectomized mice. It specifically targets Sirt1 and activates NF-κB signaling pathways (42). Xu et al. identified increased levels of miR-143/145 in the serum, saliva, and BMSCs of postmenopausal women, which impede self-renewal and differentiation. Depletion or inhibition of antagomiR-143/145 mitigated bone loss and impaired regeneration in estrogen-deficient osteoporosis (43). MiR-301b, miR-146a, and miR-148a represent prospective therapeutic targets for osteoporosis resulting from estrogen deficiency. MiR-146a safeguards against bone resorption, whereas global knockout impairs osteoclast activity (35). MiR-148a-/− mice exhibited augmented bone mass and diminished resorption in osteoporosis models, whereas depletion curtailed excessive resorption; conversely, agomiR-148a or AAV-shNRP1 accelerated the progression of osteoporosis (39). β-CTX secretion in PMOP patients correlates with miR-148a expression, whereas miR-301b is upregulated in bone tissue. The osteoclastic conditional knockout diminishes osteoclastogenesis and the quantity of osteoclasts, thereby offering substantial bone protection (39). MiR-301-b directly targets CYLD, an anti-inflammatory factor. CYLD modulates osteoclastogenesis through the activation of NF-κB signaling. *In vivo* investigations regarding osteoporosis related to lncRNA or circRNA are scarce (44). LncRNA Nron is prevalent in bone tissue and diminished in OVX mice. Osteoclasts exhibit diminished Nron expression alongside increased bone resorption. Transgenic mice or pharmaceutical overexpression of Nron resulted in increased bone mass due to inhibited osteoclastogenesis, involving Nron's interaction with CUL4B and its NCM2 motif, which regulates the Erα pathway (45)(**Figure 3**). To summarize, multiple studies have recognized ncRNAs as crucial regulators of osteoporosis. Investigations into lncRNAs and miRNAs are essential for osteogenesis and osteoclastogenesis; however, there is a paucity of studies regarding circRNA functionality in transgenic animals, which is necessary to elucidate the pathogenesis of osteoporosis. Comprehending the regulation of ncRNAs will facilitate the identification of specific targets for the diagnosis and treatment of osteoporosis. **Table 2** delineates the function of select ncRNAs in osteoporosis and their mechanisms in bone resorption.

**Table 2:** The molecular mechanisms of specific ncRNAs in osteoporosis and bone resorption.

NcRNAs	Targeting pathways	Molecular function	References
MiR-148a	NRP1	Its knockout mouse model effectively protects against excessive bone resorption in mice.	(39)
MiR-146a	RANKL/OPG signaling	The osteoclast activities were observed to be compromised in miR-146a knockout mice subjected to estrogen deficiency.	(46)
MiR-301b	CYLD/NF-κB signaling	Ablation of osteoclastic miR-301-b suppressed OVX-induced osteoclastogenesis.	(44)
MiR-128	SIRT1/NF-κB signaling	The deletion of miR-128 in osteoclasts inhibited osteoclastogenesis and provided a protective effect against bone loss.	(42)
MiR-143/MiR-145	Cd226 and Srgap2	MiR-143/145 was delivered to osteoclasts via extracellular vesicles, initiating osteoclastic activity.	(47)
MiR-21	Spry1 and PDCD4	The lack of miR-21 has been shown to impede bone resorption and osteoclast activity.	(48)
LncRNA (Nron )	CUL4B/Erα signaling	Nron knockout mice exhibit an osteopenic phenotype characterized by heightened bone resorption activity.	(43)

## 5 The regulatory function of ncRNAs in osteoarthritis

Osteoarthritis (OA) is a common condition that induces pain and disability, adversely affecting quality of life and imposing a socio-economic burden worldwide. The condition impacts the hip, knee, and finger joints, resulting in cartilage deterioration, subchondral bone alteration, and osteophyte formation, affecting more than 25% of individuals aged 18 and above (49). The majority of osteoarthritis patients have not received sufficient treatment, resulting in merely symptomatic relief. The identification of biomarkers for early diagnosis, subgroup classification, staging, and disease prognosis is imperative. Comprehending the mechanisms of pathogenic osteoarthritis will facilitate the identification of novel biomarkers to avert or postpone disease progression (50). NcRNAs are essential in cartilage development and maintenance by regulating chondrocyte proliferation, differentiation, and extracellular matrix biosynthesis. Aberrant ncRNA expression can result in ECM degradation, hypertrophy, and apoptosis, contributing to osteoarthritis. *In vivo* studies underscore the essential roles of ncRNAs in preserving cartilage homeostasis (51).

Monocytes can differentiate into two types of macrophages: M1 recruits immune cells during inflammation, while M2 suppresses immune cell secretion, promoting angiogenesis and tissue repair. The production of M1 and M2 macrophages must be balanced in order to prevent tissue damage. While M2 promotes tissue repair and inhibits the release of immune cells, cytokines such as LPS and TNF-α promote the accumulation of M1. Research indicates that PPARβ/δ agonists and pseudolactulic acid B prevent osteoporosis, while BMSC-Exo can improve osteoarthritis by regulating inflammation and macrophage polarization (52).

### 5.1 Beneficial miRNA modulators for the maintenance of articular cartilage homeostasis

Recent studies indicate that Sox9 is essential in cartilage development and the pathogenesis of osteoarthritis. MiR-140, a cartilage-specific non-coding RNA, is modulated by Sox9. MiR-140 knockout mice exhibit reduced stature and diminished body weight, yet manifest osteoarthritis-like pathology after 12 months. MiR-140-/− mice exhibit diminished articular cartilage damage in surgical arthritis models and exhibit milder osteoarthritis symptoms in antigen-induced arthritis models, highlighting its involvement in the progression of osteoarthritis (53, 54). MiR-455, an abundantly expressed RNA in chondrocytes, modulates cartilage homeostasis in both human and murine chondrocytes; however, in mice, it precipitates accelerated cartilage degeneration (55). Studies indicate that miR-455-deficient mice display osteoarthritis-like pathology at six months, whereas miR-455-3p and 5p mimics substantially hinder cartilage degeneration in mice subjected to DMM surgery. MiR-455-5p and -3p suppressed HIF-2α, a modulator of cartilage homeostasis, and siRNA-mediated silencing of HIF-2α mitigated cartilage degeneration *in vivo* (55). Runx2 modulates chondrocyte hypertrophy and the advancement of osteoarthritis, whereas miR-204 and miR-211 are microRNAs that preserve joint homeostasis by inhibiting osteoarthritis development. The global knockout of miR-204/211 induces spontaneous osteoarthritis by 15 weeks, while conditional knockout in mesenchymal progenitors displays aging-like phenotypes, and its reduction results in Runx2 accumulation and cartilage degradation. The research indicated that mesenchymal progenitor cells in dKO mice generated an overabundance of mesenchymal cells, resulting in synovial hyperplasia. Nonetheless, these osteoarthritis phenotypes may be enhanced through intra-articular administration of AAV5-miR-204 or by silencing Runx2 (46). The study also highlighted the role of certain ncRNAs in osteoarthritis. A novel lncRNA, AC005165.1, is dysregulated in osteoarthritis articular cartilage and subchondral bone, influencing the expression of the osteoarthritis risk gene FRZB, indicating potential therapeutic targets (56).

### 5.2 Negative miRNA regulators of cartilage homeostasis

Wang and associates identified the upregulation of miR-21-5p in osteoarthritis patients, resulting in the creation of cartilage-specific knockout mice that inhibit spontaneous osteoarthritis in mice at 12 months (57). The research indicated that intra-articular administration of antagomiR-21-5p mitigated articular cartilage degeneration in a murine DMM model, whereas agomiR-21-5p exacerbated the condition. FGF18 was identified as a direct target of miR-21-5p, and its protein expression was elevated in cKO mice. Mir-21-5p is associated with temporomandibular joint osteoarthritis. The study revealed that miR-21-5p−/− mice exhibited diminished TMJOA progression by expressing fewer inflammatory genes and proteins (57). MiRNA target databases identified Spry1 as a target gene of miR-21-5p, and miR-141/200c was elevated in osteoarthritis patients. The research indicated that miR-141/200c, a gene associated with cartilage repair, can mitigate OA in murine models, and its delivery via nanoparticles can impede OA progression by targeting SIRT1, a deacetylase (47). MiR-146a, a biomarker for OA, was identified in early OA patients and mice subjected to destabilization of the medial meniscus (DMM) surgery. Furthermore, in mice deficient in MiR-146a, both spontaneous OA and OA induced by knee destabilization were inhibited. Lenti-miR-146a-mimic suppressed the expression of Sox9 and Col2a1 in murine articular cartilage cells, whereas Lenti-miR-146a-inhibitor counteracted this effect, suggesting potential therapeutic advantages in osteoarthritis management (48). MiR-146a modulates cartilage anabolism by targeting genes such as Tgif1, Camk2d, and Ppp3r via NF-κB-dependent signaling. It also elevates miR-483-5p expression in the articular cartilage of osteoarthritis patients and mice with DMM-induced osteoarthritis (48). The Bai group (58) created doxycycline-inducible miR-483 transgenic mice to examine its crucial function in osteoarthritis. The research revealed that miR-483-5p, an RNA regulated by p53, modulated mTORC1, a transcription factor, and its interaction with Matn3 and Timp2 in mice promoted chondrocyte hypertrophy and cartilage angiogenesis. MiR-34a-5p, a protein associated with obesity, was observed to be elevated in the plasma, cartilage, and synovium of osteoarthritis patients and mice with DMM-induced osteoarthritis. The intra-articular delivery of miR-34a-5p or its complete knockout safeguarded cartilage from damage induced by DMM (59).

### 5.3 Circular RNAs in osteoarthritis

Recent advancements in high-throughput RNA sequencing have resulted in the identification of more than 30,000 circRNAs, with ongoing research on circRNAs in osteoarthritis continually progressing. RNA sequencing has demonstrated the upregulation of circNFKB1 and circGCN1L1 in human chondrocytes (60–62). The research indicated that the knockdown of circNFKB1 suppressed ECM catabolism and accelerated osteoarthritis progression in mice. Injection of ad-circNFKB1 into mice with DMM-induced osteoarthritis resulted in cartilage degradation and osteophyte development. CircGCN1L1 promoted chondrocyte apoptosis, and inflammation, and reduced ECM anabolism in TMJ OA by sequestering miR-330-3p and targeting TNF-α (61). CircRNAs, including circSERPINE2, circPDE4B, and circFOXO3, can mitigate osteoarthritis progression by downregulating miR-1271 and E26 transformation-specific-related genes, thereby promoting cartilage protection (63–65). The research indicated that adeno-associated virus overexpressing circSERPINE2 mitigated the osteoarthritis phenotype in rabbits by modulating chondrocyte proliferation and extracellular matrix metabolism, with the circPDE4B-RIC8A axis being pivotal in the regulation of p38 MAPK signaling (65, 66). CircFOXO3 induces autophagy, mitigates chondrocyte apoptosis, and enhances ECM anabolism. The *in vivo* anti-OA effect was confirmed, demonstrating that it downregulates CircPARD3B in synovial tissues, thereby inhibiting synovial angiogenesis through miR-326 and SIRT1 (64, 67). The research investigates the capacity of small non-coding RNAs, such as tRNA-derived fragments, as novel instruments for assessing fracture healing and tissue regeneration. Following the fracture, levels of miR-451a diminished, whereas miR-328-3p, miR-133a-3p, miR-375-3p, miR-423-5p, and miR-150-5p exhibited an increase, indicating that fracture healing may induce systemic metabolic alterations (68). Functional studies will confirm their potential in therapeutic applications aimed at fracture healing processes or as biomarker tools, utilizing circulating small non-coding RNAs as indicators.

### 5.4 LncRNAs in osteoarthritis

**Figure 4:**
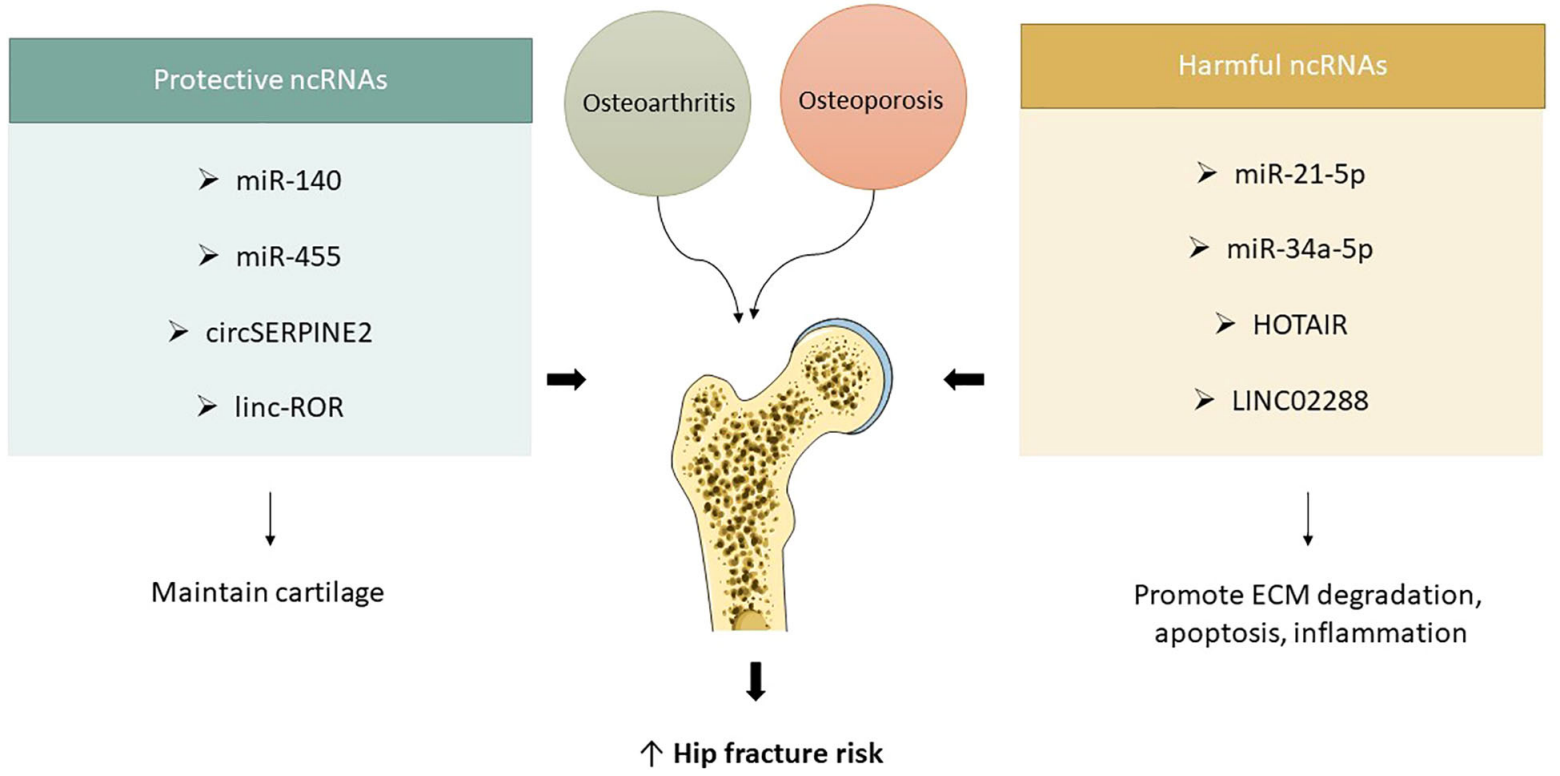
ncRNAs in osteoarthritis and hip fracture vulnerability. Protective ncRNAs (e.g., miR-140, miR-455, circSERPINE2, linc-ROR) help maintain cartilage structure and joint integrity, while harmful ncRNAs (e.g., miR-21-5p, miR-34a-5p, HOTAIR, LINC02288) promote cartilage breakdown, inflammation, and apoptosis. Their dysregulation links osteoarthritis progression with increased fracture risk in osteoporotic hips.

lncRNAs, which do not translate into polypeptides, are essential in the regulation of gene expression. They are frequently dysregulated in cartilage or synovial fluid during the pathogenesis of osteoarthritis. Recent studies indicate that HOTAIR and LINC02288 are upregulated in human osteoarthritic cartilage, with LINC02288 facilitating chondrocyte apoptosis and inflammation through the modulation of the miR-347a-3p/RTN axis (69, 70). HOTAIR, a protein, interacts with miR-17-5p and enhances FUT2 expression, resulting in chondrocyte apoptosis and extracellular matrix degradation. This axis facilitates the progression of osteoarthritis through the Wnt/β-catenin pathway. Intra-articular injections cause significant cartilage deterioration (71). HOTAIR suppressed chondrocyte proliferation, facilitating apoptosis and extracellular matrix degradation via the miR-20b/PTEN pathway, whereas MM2P safeguarded chondrocyte differentiation, augmented Colla2 and Acan expression, and stimulated proteoglycan and type II collagen secretion in chondrocytes by inducing M2 macrophage polarization and enhancing the transfer of M2-derived exosomal SOX9 (72). Linc-ROR, a long non-coding RNA, enhances chondrogenesis and cartilage formation in mesenchymal stem cells by modulating SOX9 expression. It additionally acts as a sponge for miR-138 and miR-145, inhibiting the chondrogenic activity of BMSCs and the expression of SOX9. The co-expression of linc-ROR demonstrates a restorative effect. These ncRNAs have been validated in transgenic murine models (73) (**Figure 4**).

### 5.5 LncRNAs in hip fracture

An orthopedic condition that is frequently caused by trauma, tumor excision, and other abnormalities is skeletal fracture. Local soft tissue, vascular damage, and a loss of bone mechanical integrity are all involved. A sophisticated network of cells and cytokines works to repair broken bones and restore skeletal function during fracture healing. In order to sustain osteoblast differentiation and activity, hormones, growth factors, and cytokines that control gene expression are essential. Due to high-energy traffic injuries, femoral neck fractures are more common in young or middle-aged people. Although lncRNAs play a significant role in carcinogenesis, their function in osteogenic differentiation has not received much attention (74, 75). According to a study, SNHG7 may function as a biomarker because it is down-regulated in femoral neck fracture tissues and correlated with fracture type. Osteoblast cell migration and proliferation were inhibited by SNHG7 knockout, while cell apoptosis was increased and osteoblastic activity was reduced when SNHG7 was silenced. This emphasizes how crucial it is to comprehend lncRNAs in relation to skeletal fractures. Potential targets for microRNAs binding with SNHG7 were predicted by a bioinformatics analysis, and studies indicate that lncRNAs can modulate miRNA (76). SNHG7 was found to target miR-9, which may be involved in osteoblast differentiation and bone formation. Fractured tissues had higher levels of miR-9, indicating that miR-9 inhibitors may be able to inhibit the growth of si-SNHG7 cells. LncRNAs are involved in lncRNA-miRNA-mRNA crosstalk and ceRNA networks. By attaching itself to the 3'-UTR, MiR-9 has been shown to suppress the expression of TGFBR2. Inhibition of TGFβ signaling can hinder osteoblast migration and differentiation during fracture healing, as TGFβ is essential for the regulation of osteoblasts and osteoclasts (77). According to the study, TGFBR2 expression was downregulated in MC3T3-E1 cells when miR-9 was overexpressed, and TGFBR2 levels were lower in fractured tissues. The expression of TGFBR2, p-smad2, and p-smad3 in cells was markedly reduced by SNHG7 knockout. The complicated process of fracture healing controls the activation, growth, and differentiation of progenitor cells, also known as local mesenchymal stem cells. However, delayed healing or nonunion occurs in 5–10% of fracture patients. The goal of research is to prevent fracture nonunion and increase fracture healing rates. With HAGLR downregulated in fractured femoraneck tissues and linked to the type of bone fracture, lncRNAs are essential for fracture healing (78, 79). Inhibition of HAGLR resulted in decreased osteoblast activity, apoptosis, migratory capacity, and viability. The inhibitory effects of miRNA on downstream genes are lessened by lncRNA, which sponges’ miRNA to suppress its expression. H19 sponges use the Wnt pathway to speed up osteogenesis in hMSCs by upregulating RUNX2 through miRNA-141 and miR-22. In tissues from fractured femoral necks, miRNA-19a-3p is upregulated and may be a target of HAGLR (79). Cell division, embryogenesis, and bone remodeling are all inhibited by TGF-β signaling. TGFBR2 is downregulated in femur bone fractures, and MC3T3-E1 cells' TGFBR2 level is inhibited by miRNA-19a-3p overexpression. TGFBR2, p-smad2, p-smad3, and RUNX2 were all downregulated in MC3T3-E1 cells through HAGLR knockdown. The TGF-β pathway is inhibited by downregulating HAGLR, which speeds up the healing of femur neck fractures. The research examines the function of LncRNA NR2F1-AS1 in osteogenic differentiation, employing an *in vitro* model and real-time fluorescence quantitative PCR to identify osteogenic differentiation markers. Cell proliferation and apoptosis were evaluated using DLR assay, RIP, and ELISA, validating gene interactions and measuring alkaline phosphatase activity. Bioinformatics methodologies forecasted genes and pathways, indicating that OS augments osteogenic differentiation markers and NR2F1-AS1 expression, whereas the suppression of miR-423-5p promotes osteoblast differentiation, stimulates proliferation, activates ALP activity, and inhibits apoptosis (78, 80, 81).

## 6 The application of lncRNAs and cirRNAs in osteoporosis diagnosis and prognosis

The application of circRNAs and lncRNAs in the prediction and diagnosis of osteoporosis is constrained by a paucity of studies involving human subjects. Chen et al. (82) discovered elevated levels of lncRNA XIST in the peripheral blood monocytes of osteoporosis patients, whereas Fei et al. identified a significant downregulation of 51 lncRNA transcripts in this population. The mRNAs of postmenopausal women were associated with inflammatory response, osteoclast differentiation, and cytokine-cytokine receptor interaction (83). ALP, which is downregulated in postmenopausal women, was located within 100 kb of lncRNAs, suggesting a cis-regulatory influence of lncRNAs on mRNAs associated with bone metabolism. The study comprised three women and two healthy controls. Tong et al. discovered that monocytes in postmenopausal women exhibit elevated levels of lncRNA DANCR, indicating its potential as a biomarker for osteoporosis, particularly as monocytes from women with diminished bone mineral density show increased expression of this RNA (84). Monocytes that are overexpressed exhibit elevated levels of TNF-α and IL-6, which are associated with osteoporosis. DANCR expression correlates with low bone mineral density osteoporosis. Anti-IL-6 or anti-TNF-α therapies may mitigate elevated bone-resorbing activity in murine bone cultures. The association between DANCR, IL-6, and TNF-α in osteoporosis therapies remains under examination. The limited concentration of lncRNAs in plasma/serum may impede their analysis, potentially postponing their application as cell-free biomarkers for diagnosis and prognosis (85, 86). A study identified XIST upregulation in patients' serum, underscoring the difficulties in diagnosing osteoporosis through lncRNAs in plasma or serum. The increased expression of CASC11, a crucial diagnostic marker for osteoporosis, was associated with an extended treatment duration and a high recurrence rate. Plasma SNHG1, a downregulated lncRNA in postmenopausal women, exhibited significant downregulation, especially in those with PMOP, positioning it as a potential biomarker for PMOP diagnosis (87). CircRNAs are non-coding RNA molecules comprising hundreds of nucleotides, categorized into exon circular RNAs, intronic circRNAs (ciRNAs), and exon-intron circular RNAs (EIciRNAs). They employ mechanisms such as peptide transformation, protein interaction, and miRNA sponges for physiological and pathological processes. Research indicates that the highly expressed circRNA Circ_0002060 may function as a diagnostic biomarker for osteoporosis, exhibiting 78% sensitivity and 69% specificity in clinical samples. Its concentration is associated with diminished bone mineral density and T-score, but not with weight, height, or age. Circ_0006393, conversely, exhibits low expression levels and facilitates bone remodeling (88).

## 7 The application of lncRNAs and cirRNAs in osteoporosis treatment

lncRNAs, which regulate bone metabolism, are being investigated as potential target molecules for novel osteoporosis therapies. Nonetheless, their implementation in clinical settings remains nascent. Only 25 clinical trials are registered, primarily concentrating on validating lncRNAs as biomarkers for diagnosis and prognosis, rather than as therapeutic agents. These trials primarily concentrate on cancer and cardiovascular patients, with no assessments conducted on individuals with musculoskeletal disorders. The therapeutic utilization of lncRNAs is impeded by insufficient understanding of their biological functions and the limitations of conventional gene therapies. These factors encompass low efficacy of *in vivo* transgene transfection, repeated utilization of immunogenic gene delivery vehicles, and erratic transgene behavior, frequently resulting in malignancies (89, 90). Innovative strategies to modulate lncRNA expression *in vivo* are being developed to address these challenges, with potential applicability to other conditions such as osteoporosis. Sidi et al. (91) employed the BC-819 plasmid to treat bladder cancer patients by overexpressing the H19 promoter and regulating a toxin, thereby enhancing transfection and administering it into the bladder. The research demonstrated that a hybrid system combining a sleeping beauty transposon and baculovirus, which induces cell apoptosis and suppresses proliferation, effectively hinders cell proliferation in a mouse model of hepatocellular carcinoma. A system for the overexpression of MEG3 lncRNA was established utilizing MS2 bacteriophage virus-like particles cross-linked with the GE11 polypeptide. This approach successfully suppressed tumor proliferation in a murine model of hepatocellular carcinoma. Hu et al. (92) employed liposome-based invivofectamine® to diminish MALAT1 expression in chemoresistant prostate cancer. They employed functionalized carbon nanotubes to administer anti-MALAT1 antisense oligonucleotides, thereby diminishing tumor burden. A study demonstrated the downregulation of lncRNA KCNQ1OT1 in murine models, emphasizing cardiotoxicity. Lentiviruses incorporating shRNA diminished lncRNA levels, influencing cardiotoxicity. Strategies for LncRNA knockdown are contingent upon localization, the efficacy of synthetic nucleic acids, and the duration of effect. Single-stranded oligonucleotides are favored (93).

Short hairpin RNAs (shRNAs) are double-stranded DNA entities delivered as double-stranded DNA constructs within plasmids. They are transcribed and processed within target cells, yielding small RNA molecules with a defined secondary structure. ShRNAs can be continuously transcribed, yielding an extended therapeutic effect and minimizing off-target gene effects. The overexpression of lncRNA poses technical challenges and controversies; however, advancements in siRNA and miRNA facilitate this process (94, 95). Overexpression necessitates vectors and delivery systems with enhanced transfection efficiency. *In vitro* transfection serves as an alternative to *in vivo* cell transfection, and lncRNAs are found within extracellular vesicles. EVs can be directed towards particular cell types and modified to incorporate specific molecules, rendering them as targeted delivery vehicles for tissues (96). Mesenchymal stem cells and osteoclasts can internalize extracellular vesicles from various sources, influencing osteogenesis and osteoclastogenesis, indicating that extracellular vesicles may serve as carriers for long non-coding RNAs implicated in the regulation of bone metabolism. The overexpression or downregulation of lncRNA *in vivo* can be mitigated by the capacity of lncRNAs to recruit and bind proteins such as PCR2189 and PUMILIO190 (97, 98). This implies that their activity may be modulated by compounds capable of binding to target lncRNAs in a similar manner. Research should concentrate on identifying pharmaceuticals that can target lncRNAs, including small molecules or structurally analogous decoy proteins. The inherent interaction of lncRNAs with proteins and ligands may be investigated as carrier-like entities for drugs and proteins, including those for osteoporosis therapy (98). Advancements in genome editing, especially CRISPR/Cas9 technology, have facilitated the permanent regulation of lncRNA expression in human cells at the genetic level. This method broadens the potential for modulating lncRNA expression through strategies primarily aimed at RNA. The inaugural clinical trial in human subjects to assess the safety and efficacy of this method has commenced, with the initial trial registered under USA approval in August 2018. Chen et al. (99) investigated LncRNA gene editing via CRISPR/Cas9 *in vitro* to elucidate the enhancer role of the genomic locus rs6426749 concerning lncRNA LINC00339. The complex structure of lncRNAs may promote RNAi-based therapeutic approaches for lncRNAs, owing to enhanced specificity and diminished risk of gene disruption (100).

## 8 The role of ncRNAs in osteoporosis in relation to hip fractures

Peptidoglycan recognition protein 1 (PGLYRP1) is a crucial mRNA in osteoporosis, a condition characterized by the dysregulation of innate immunity and bacteriostatic activity. It contributes to the negative regulation of interferon-gamma production and the innate immune response. The differentiation of osteoclasts is stimulated by reduced expression of solute carrier family 11 member 1 (SLC11A1), which also plays a role in the positive regulation of interferon-gamma production and the homeostasis of cellular cadmium ions. PGLYRP1 and SLC11A1 may influence the advancement of osteoporosis by regulating various biological processes. The C-C type chemokine (CCR3) significantly contributes to osteoporosis by interacting with various chemokines and elevating intracellular calcium ion levels. CCR3 may promote osteoclast migration and is elevated during osteoclastogenesis. Osteoporosis is associated with sarcopenia, a condition predominantly connected to metabolic disorders including diabetes, obesity, and cachexia. These conditions demonstrate analogous biological pathways and risk factors, increasing bone vulnerability to osteoporosis and augmenting the probability of falls attributable to sarcopenia (101). A study aimed to clarify the RNA sequencing profile of sarcopenia in elderly female patients with osteoporosis and hip fractures, identifying a significant genetic candidate implicated in the etiology of hip fractures. The research indicates that miR-23b-3p and miR-140-3p, in conjunction with miR-21-5p, miR-122-5p, and miR-125b-5p, are associated with osteoporotic hip fractures. These miRNAs are involved in bone metabolism by suppressing osteoblast expression and facilitating osteoclastogenesis. MiR-21-5p and miR-125b-5p are associated with osteoblastogenesis and osteoclastogenesis, whereas miR-122-5p is downregulated in osteoporosis. The function of these miRNAs in bone metabolism is still ambiguous. Peripheral blood samples were collected from healthy controls, osteoporosis patients without vertebral fractures, and patients with vertebral fractures (102–105). Data were consolidated to identify differentially expressed mRNAs and lncRNAs. Protein-protein interaction networks were established, and enrichment analyses were conducted. The Cytoscape-cytoHubba plug-in was employed to identify essential differentially expressed mRNAs. A total of 3,378 lncRNA-mRNA pairs were identified, with co-expressed mRNA predominantly enriched in immune-related pathways. This study indicates that miR-23b-3p and miR-140-3p, in conjunction with miR-21-5p, miR-122-5p, and miR-125b-5p, are associated with osteoporotic hip fractures. These miRNAs are involved in bone metabolism by suppressing osteoblast expression and facilitating osteoclastogenesis. MiR-21-5p and miR-125b-5p are associated with osteoblastogenesis and osteoclastogenesis, whereas miR-122-5p is downregulated in osteoporosis. The function of these miRNAs in bone metabolism is still ambiguous (105, 106).

## 9 Conclusion and future outlooks

Multiple *in vivo* studies have demonstrated that ncRNAs are essential in skeletal development and disorders through the regulation of gene expression. Identifying essential ncRNAs among the myriad of ncRNAs present in skeletal tissues poses a challenge in elucidating their interactions. Tissue-specific ncRNAs are essential *in vivo*, while *in vitro* investigations have elucidated their direct targets and roles in particular cell types. *In vivo*, functional validation can elucidate the roles of these ncRNAs within the physiological system. However, the investigation of ncRNAs as biomarkers and therapeutic targets for osteoporotic hip fractures remains nascent, necessitating further research on biological mechanisms, detection methodologies, and *in vivo* therapeutic administration. Nonetheless, recent findings and technological innovations are encouraging for the treatment of osteoarthritis, osteoporosis and related hip fractures.
